# Unravelling the heterogeneity of oral squamous cell carcinoma by integrative analysis of single‐cell and bulk transcriptome data

**DOI:** 10.1111/jcmm.18108

**Published:** 2024-01-26

**Authors:** Jia Li, Shengjiao Li, Mingyang Shu, Weiwei Hu

**Affiliations:** ^1^ Department of Prosthodontics Shanghai Engineering Research Center of Tooth Restoration and Regeneration Stomatological Hospital and Dental School of Tongji University Shanghai China; ^2^ Department of Oral and Maxillofacial Surgery Shanghai Engineering Research Center of Tooth Restoration and Regeneration Stomatological Hospital and Dental School of Tongji University Shanghai China; ^3^ Department of Stomatology Huai'an Second People's Hospital and The Affiliated Huai'an Hospital of Xuzhou Medical University Huai'an China

**Keywords:** cellular heterogeneity, integrin‐MAPK signalling, oral squamous cell carcinoma (OSCC), scRNA‐seq, tumour microenvironment (TME)

## Abstract

Oral squamous cell carcinoma (OSCC) is a prevalent malignancy of the head and neck with rising global incidence. Despite advances in treatment modalities, OSCC prognosis remains diverse due to the complex molecular and cellular heterogeneity within tumours, as well as the heterogeneity in tumour microenvironment (TME). In this study, we utilized single‐cell RNA sequencing (scRNA‐seq) analysis to explore distinct subpopulations of tumour cells in OSCC tissues and their interaction with components in TME. We identified four major tumour cell subpopulations (C0, C1, C2 and C3) with unique molecular characteristics and functional features. Pathway enrichment analysis revealed that C0 primarily expressed genes involved in extracellular matrix interactions and C1 showed higher proliferation levels, suggesting that the two cell subpopulations exhibited tumour aggressiveness. Conversely, C2 and C3 displayed features associated with keratinization and cornified envelope formation. Accordingly, C0 and C1 subpopulations were associated with shorter overall and disease‐free survival times, while C2 and C3 were weakly correlated with longer survival. Genomic analysis showed that C1 demonstrated a positive correlation with tumour mutation burden. Furthermore, C0 exhibited resistant to cisplatin treatment, while C1 showed more sensitive to cisplatin treatment, indicating that C0 might exhibit more aggressive compared to C1. Additionally, C0 had a higher level of communication with fibroblasts and endothelial cells in TME via integrin‐MAPK signalling, suggesting that the function of C0 was maintained by that pathway. In summary, this study provided critical insights into the molecular and cellular heterogeneity of OSCC, with potential implications for prognosis prediction and personalized therapeutic approaches.

## 1 INTRODUCTION

Oral squamous cell carcinoma (OSCC) is a prevalent malignant tumour of the head and neck, characterized by clinical manifestations such as oral ulcers, neck lymph node enlargement, gingival swelling and loose teeth.^1^ The global incidence of OSCC is on the rise, with variations depending on geographical regions and populations.^2^ Timely diagnosis and appropriate treatment are pivotal in improving the prognosis of OSCC, as it is influenced by multiple factors, including tumour size, location, differentiation degree, as well as the patient's age, health status and treatment response.^2^ Treatment options for OSCC encompass surgical resection, radiation therapy, chemotherapy and targeted therapy, with the treatment plan tailored to individual patient circumstances. Due to the heterogeneity, OSCC can be classified into different pathological types and clinical stages, which significantly impact the disease's prognosis.^3^

Tumour heterogeneity in OSCC has significant clinical implications, as it can influence disease progression, treatment response and patient outcomes.^4^, ^5^, ^6^, ^7^ Recently, single‐cell analysis has emerged as a valuable tool in OSCC research, enabling the identification of molecular and cellular heterogeneity of OSCC tumour cells and the complexity of TME. Recent studies utilizing single‐cell RNA sequencing and spatial transcriptomics have identified distinct cell populations and gene expression patterns associated with OSCC precancerous lesions, offering crucial insights into early cancer development and uncovering novel targets for cancer prevention and treatment.^8^ Furthermore, single‐cell analysis sheds light on the tumour microenvironment, revealing interactions between tumour cells, immune cells and stromal cells. Single‐cell RNA sequencing has been employed to analyse the immune microenvironment of OSCC, highlighting distinct tumour cell subpopulations with different molecular profiles and functional attributes, thereby fostering the development of personalized treatment strategies for this intricate disease.^9^ In addition, integrative single‐cell and bulk transcriptome analyses have unveiled three intrinsic subtypes of OSCC, each with distinct prognoses and therapeutic vulnerabilities, further supporting the potential of personalized treatment approaches for OSCC patients.^10^ Additionally, a recent study has identified immunostimulatory cancer‐associated fibroblast subpopulations that predict immunotherapy response in head and neck cancer, which provides critical insights into the tumour microenvironment and may promote the development of new biomarkers for predicting treatment response and improving patient outcomes.^11^ These studies collectively demonstrate the power of single‐cell transcriptomic analyses in understanding the molecular and cellular heterogeneity and the complexity of TME of OSCC and in devising targeted therapeutic interventions. In order to elucidate the diversity of tumour cell subpopulations in OSCC and their associations with clinical characteristics and tumour microenvironment composition, we conducted single‐cell RNA‐seq data analysis. Through this analysis, we identified highly malignant tumour cell subpopulations and potential mechanisms underlying the functional maintenance of these subpopulations.

## 2 MATERIALS AND METHODS

### 2.1 Single‐cell RNA sequencing (scRNA‐seq) analysis

ScRNA‐seq data from three pairs of tumour tissues collected before and after nivolumab treatment were collected from the previous research,^11^ and were utilized to identify distinct subpopulations of tumour cells. The scRNA‐seq analysis was implemented in R/Seurat package.^12^ Specifically, count table data were initially subjected to quality control measures, including the removal of low‐quality cells and genes based on read counts (between 500 and 5000) and mitochondrial gene content (<20%). Normalization techniques were then applied to account for variations in sequencing depth and library size, followed by gene selection to filter out low‐expressed genes. Subsequently, dimensionality reduction techniques such as principal component analysis (PCA) were employed to identify significant sources of variation, and graph‐based clustering algorithm (Louvain algorithm) was utilized to group the single cells into clusters using the top 20 principal components. Cell types were assigned to clusters through marker gene identification and annotation. The integration of data from multiple batches was achieved using the Harmony tool to mitigate batch effects.^13^ Differential expression analysis was performed to identify genes showing differential expression between cell types (Table S1), and t‐distributed Stochastic Neighbor Embedding (t‐SNE) was employed for visual exploration.

### 2.2 Cell cycle score

The cell cycle scores for the cancer cells were calculated by R Seurat CellCycleScoring. Briefly, the Seurat object of cancer cells and gene list of G2M phase and S phase were used as input. The scoring strategy employed the method of an early study.^14^

### 2.3 Bulk gene expression data

The bulk gene expression data and clinical data of TCGA OSCC cohort were downloaded from UCSC Xena database.^15^ The expression levels were transformed to log2 (FPKM+1). The microarray‐based gene expression data were downloaded from ArrayExpress with accession E‐MTAB‐8588.^16^ Feature intensities of the microarray data were background corrected and quantile normalized in R package limma. Intensity values are in log2 scale. OSCC samples were extracted based on the previous study.^17^ The cell line gene expression data were extracted from Gene Expression Omnibus (GEO) with accession GSE168424. Specifically, two human OSCC cell lines (H103 and SAS) each representing distinct tumour staging, along with an in vitro cisplatin‐resistant model derived from the cancer stem cells (CSCs) of the SAS cell line, were employed. The gene expression levels were computed using the Robust Multi‐array Average (RMA) algorithm, quantile‐normalized and log2‐transformed.

### 2.4 Re‐clustering of cancer cells

After the initial analysis, cancer cells were re‐clustered to discover four different tumour cell subgroups (C0, C1, C2 and C3). Specifically, the nearest‐neighbour graph of cancer cells was re‐constructed the harmony‐based reduction. The graph‐based clustering algorithm (Louvain algorithm) was utilized to group the cancer cells into subclusters with a resolution at 0.2.

### 2.5 Differential gene expression analysis

The bulk gene expression data were initially log2‐normalized and were subsequently processed by R/limma package.^18^ Specifically, a design matrix is constructed to define experimental conditions. Linear models are fitted to the log2‐normalized data based on the design matrix. Statistical testing generates moderated t‐statistics for each gene, facilitating the assessment of differential expression between conditions. To control false positives, the multiple testing correction method (Benjamini–Hochberg procedure) was implemented, enabling the identification of significant genes based on adjusted *p*‐values and fold change thresholds.

### 2.6 Survival analysis

We employed Cox proportional hazard regression analysis to assess the predictive potential of variables for disease‐free survival (DFS) and overall survival (OS) in patients with oral squamous cell carcinoma (OSCC). The OSCC patient cohort was stratified into high‐ and low‐risk groups based on the median value of the variables used as a threshold. To determine survival outcomes, Kaplan–Meier (KM) analysis was conducted. The KM survival curves were generated using the R survminer package (https://cran.r‐project.org/web/packages/survminer/index.html).

### 2.7 Pathway enrichment analysis

Pathway enrichment analysis of differentially expressed genes was conducted to understand the functional characteristics using R/clusterProfiler.^19^ Specifically, statistical enrichment analysis is performed using hypergeometric test to identify whether specific gene sets are overrepresented within the gene list compared to what would be expected by chance. The *p*‐values were adjusted by Benjamini–Hochberg procedure to control false discovery rate.

### 2.8 Single‐sample gene set enrichment analysis (ssGSEA)

ssGSEA scores were calculated based on the representative genes of the four cancer cell subpopulations to assess the enrichment of specific gene sets for the tumour samples using bulk gene expression data. This analysis was implemented in R/GSVA package.^20^

### 2.9 Pearson correlation analysis

Pearson correlation analysis was used to examine the association between various clinical variables and ssGSEA scores of tumour cell subpopulations, as well as the correlation between point mutations or copy number variations (CNVs) and the ssGSEA scores of each tumour cell subpopulation.

### 2.10 Clustering of genes and tumour samples based on somatic copy number alteration (sCNA) profile

The rows and columns of sCNA matrix indicated the genes and tumour samples, respectively. The copy number gain, deletion and neutral were represented by 1, −1 and 0, respectively. Genes and tumour samples were clustered based on sCNA profiles to identify distinct sCNA gene sets (G1‐G5) and classify tumour samples into two groups (S1 and S2).

### 2.11 CellChat analysis

CellChat was used to establish a regulatory network between tumour cell subpopulations and various cell types in the tumour microenvironment to explore their intercellular communication patterns.^21^ The ligand–receptor pairs annotated as ‘Secreted Signaling’ and ‘ECM‐Receptor’ were used for this analysis. Notably, the receptors with highly expressed between the cancer cell subpopulations were retained.

## 3 RESULTS

### 3.1 Single‐cell RNA‐seq analysis of tumour cell subpopulations in oral squamous cell carcinoma

**FIGURE 1:**
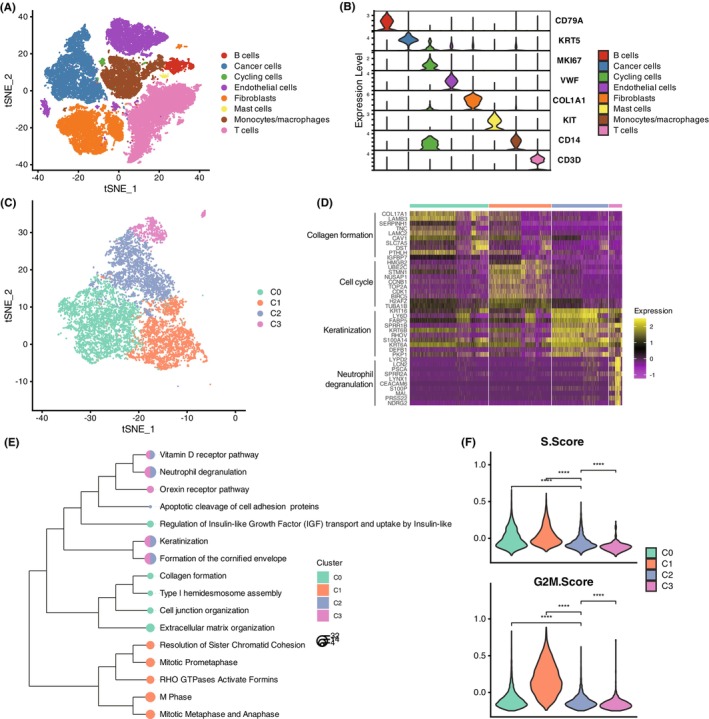
Characterization of Tumour Cell Subpopulations in OSCC. (A,B) Single‐cell transcriptomic analysis of OSCC tumour tissues pre‐ and post‐nivolumab treatment identified eight major cell types: B cell (CD79A), cancer cell (KRT5), cycling cell (MKI67), endothelial cell (VWF), fibroblast (COL1A1), mast cell (KIT), monocyte/macrophage (CD14) and T cell (CD3D). (C) Re‐clustering of cancer cells revealed four distinct tumour cell subpopulations: C0, C1, C2 and C3. (D) Differential expression analysis of genes in the four tumour cell subpopulations highlighted significant expression patterns of top 10 DEGs. (E) Pathway enrichment analysis for each subpopulation showed that C0 expressed genes related to collagen formation, hemidesmosome assembly and extracellular matrix organization. C1 exhibited high proliferation levels with enrichment in cell cycle pathways. C2 and C3 shared features related to keratinization and cornified envelope formation. (F) The S score and G2M score confirmed higher proliferation in C1 compared to other subpopulations. The symbol ****indicated *p* < 0.0001.

To explore distinct subpopulations of tumour cells in OSCC, we first utilized single‐cell transcriptomic data from three pairs of tumour tissues collected before and after nivolumab treatment. Through this analysis, we identified eight major cell types, namely B cell (CD79A), cancer cell (KRT5), cycling cell (MKI67), endothelial cell (VWF), fibroblast (COL1A1), mast cell (KIT), monocyte/macrophage (CD14) and T cell (CD3D) (Figure 1A,B, Table S1). Subsequently, we performed re‐clustering of the cancer cells and discovered four different tumour cell subpopulations, designated as C0, C1, C2 and C3 (Figure 1C, Table S2). Analysis of the differentially expressed genes (DEGs) in these four tumour cell subpopulations revealed highly significant expression patterns of the top 10 DEGs across the four cell subpopulations (Figure 1D, Table S3). Specifically, the top 10 genes of the four subpopulations were primarily involved in collagen formation (*COL17A1*, *LAMB3*, *SERPINH1*, *LAMC2* and *DST*), cell cycle (*UBE2C*, *CCNB1*, *CDK1* and *BIRC5*), keratinization (*KRT16*, *SPRR1B*, *KRT6B*, *KRT6A* and *PKP1*) and neutrophil degranulation (*LCN2*, *CEACAM6* and *S100P*). Further pathway enrichment analysis of DEGs for each cell subpopulation revealed that C0 primarily expressed genes involved in collagen formation, type I hemidesmosome assembly, cell junction organization and extracellular matrix organization‐related genes (Figure 1E), indicating a mesenchymal phenotype of this cell subpopulation.^9^ C1 showed significantly higher proliferation levels, as its DEGs were enriched in cell cycle‐related pathways such as mitotic prometaphase, M phase, mitotic metaphase and anaphase (Figure 1E). Conversely, C2 and C3 displayed similar features, including keratinization and formation of the cornified envelope, among others (Figure 1E). Consistent with the results of pathway enrichment analysis, both the S score and G2M score of C1 were higher than the other cell subpopulations (Figure 1F). Those findings indicated that single‐cell RNA‐seq analysis can accurately cluster tumour cell subpopulations with distinct molecular characteristics. These results indicated that C0 and C1 exhibited highly malignant phenotypes.

### 3.2 Clinical characteristics of cancer cell subpopulations

**FIGURE 2:**
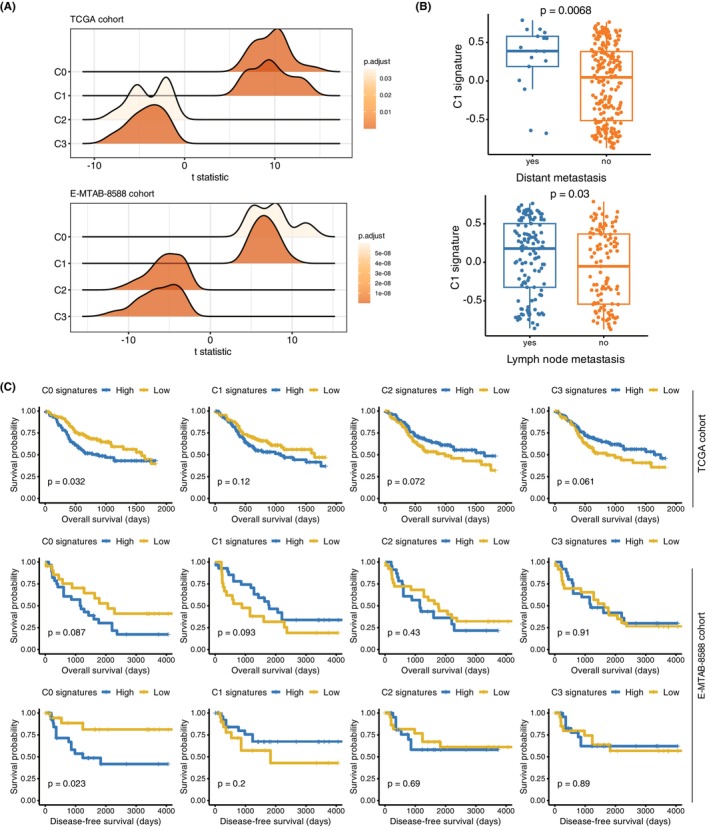
Correlation Between Tumour Cell Subpopulations and Clinical Characteristics. (A) Analysis of single‐sample gene set enrichment (ssGSEA) scores based on DEGs of the four tumour cell subpopulations (C0‐C3) revealed that representative genes of C0 and C1 were upregulated in tumour tissue, while those of C2 and C3 were downregulated, indicating potential pro‐tumorigenic roles for C0 and C1. The t statistic was calculated by R/limma, which was used to evaluate the differential expression level between OSCC and normal tissues. (B) Elevated ssGSEA scores of the C1 subpopulation were observed in primary tumours with distant metastasis and lymph node metastasis, suggesting a specific involvement of C1 in metastatic processes. The *p*‐values were calculated by Student's *t*‐test. (C) KM curves for OSCC samples with high and low ssGSEA scores of tumour cell subpopulations (C0‐C3) in TCGA and E‐MTAB‐8858 cohorts. The *p*‐values were calculated by log‐rank test.

In order to explore the correlation between various tumour cell subpopulations and clinical characteristics, we used different statistical methods to examine the association between different clinical variables and tumour cell subpopulations. Firstly, we calculated single‐sample gene set enrichment (ssGSEA) scores for the tumour samples based on the DEGs of four tumour cell subpopulations (see Materials and Methods). Specifically, we found that the representative genes of C0 and C1 were significantly upregulated in the tumour tissue, while the representative genes of C2 and C3 were relatively downregulated in the tumour tissue (Figure 2A), further suggesting that C0 and C1 might play pro‐tumorigenic or progressive roles in OSCC. On the contrary, the downregulation of representative genes in C2 and C3 subpopulations in tumour tissues suggested that these two subpopulations might be relatively rare in the tumour tissue or their role in tumour development could be limited. Upon further analysis, we observed that the ssGSEA scores of the C1 subpopulation significantly increased in the primary tumours of TCGA samples that had undergone distant metastasis and lymph node metastasis, suggesting that the C1 subpopulation of tumour cells may be specifically involved in the metastatic processes, potentially contributing to lymph node and distant metastasis (Figure 2B). Additionally, in the survival analysis of the four tumour cell subpopulations (C0‐C3) in the TCGA and E‐MTAB‐8858 cohorts, we found that C0 and C1 were correlated with shorter overall and disease‐free survival times in both of the cohorts (Figure 2C). Notably, C0 exhibited a more significant association with adverse prognosis (Figure 2C, log‐rank test, *p* < 0.05). On the other hand, C2 and C3 showed a weak correlation with longer survival times in the TCGA cohort (Figure 2C, log‐rank test, *p* < 0.1). The findings suggested that the identified tumour cell subpopulations had clinical implications and may serve as potential prognostic indicators in cancer patients.

### 3.3 Cancer cell subpopulations are associated with the sensitivity to cisplatin treatment

**FIGURE 3:**
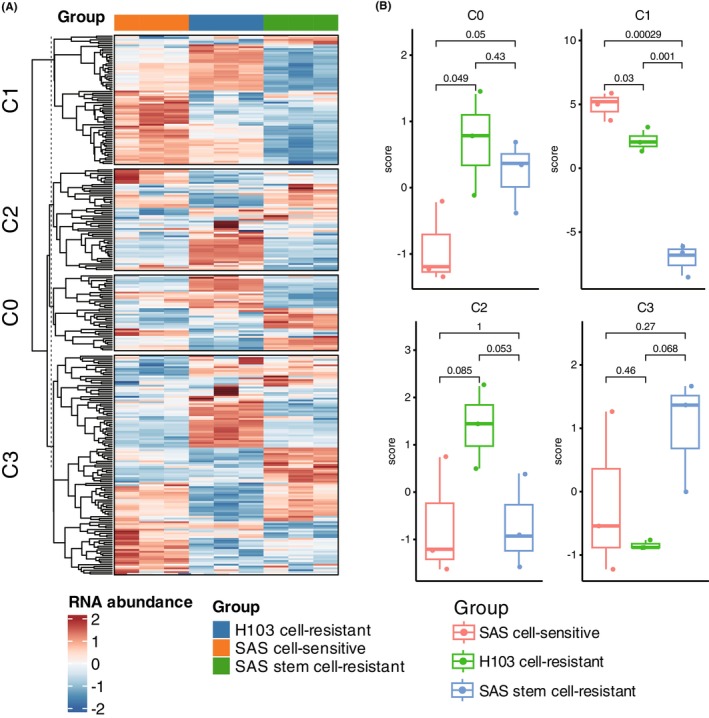
Tumour Cell Subpopulations and Cisplatin Sensitivity. (A) Comparison of DEG expression levels among three tumour cell lines (H103 drug‐resistant, SAS sensitive and SAS tumour stem cells) for the four tumour cell subpopulations revealed distinct expression patterns. (B) Calculation of ssGSEA scores across the tumour cell subpopulations for nine OSCC cell line samples demonstrated higher scores of C0 in drug‐resistant cell lines and higher scores of C1 in sensitive cell lines (*t*‐test, *p* < 0.05). C2 and C3 ssGSEA scores showed no significant differences among cell lines, reaffirming their lack of association with cisplatin sensitivity. The *p*‐values were calculated by Student's *t*‐test.

In order to investigate the relationship between the four tumour cell subpopulations and sensitivity to cisplatin treatment, we compared the expression levels of DEGs in these subpopulations among three tumour cell lines, including the drug‐resistant H103 cells, sensitive SAS cells and drug‐resistant SAS tumour stem cells. We observed that the DEGs of C0 exhibited two distinct expression modules, which were upregulated in the two drug‐resistant cell lines compared to the sensitive cell line, respectively (Figure 3A). On the other hand, the DEGs of C1 were downregulated in SAS tumour stem cells, and most of them were upregulated in the sensitive cell line (Figure 3A). Furthermore, the DEGs of C2 and C3 were uniformly distributed among the three cell lines, suggesting no significant association with cisplatin sensitivity.

Similarly, we calculated the ssGSEA scores for the nine OSCC cell line samples across the four tumour cell subpopulations. We found that the ssGSEA scores of C0 were significantly higher in drug‐resistant cell lines compared to sensitive ones, while the ssGSEA scores of C1 were significantly higher in sensitive cell lines compared to drug‐resistant ones (Figure 3B, *T*‐test, *p* < 0.05). Additionally, the ssGSEA scores of C2 and C3 showed no significant differences among different cell lines, further indicating their lack of association with cisplatin sensitivity. Considering that C0 and C1 cancer cell subpopulations were characterized by mesenchymal and proliferative phenotypes, respectively, we speculated that mesenchymal‐like cancer cells might exhibit resistance to chemotherapy, whereas proliferative cancer cells might display sensitivity to chemotherapy. Overall, these findings suggested that the specific tumour cell subpopulations (C0 and C1) have distinct gene expression patterns and are associated with differential sensitivity to cisplatin treatment, which highlighted the importance of considering the heterogeneity of cancer cell populations when studying drug responses and potential therapeutic strategies.

### 3.4 The molecular features of tumour cell subpopulations are correlated with their genomic variations

**FIGURE 4:**
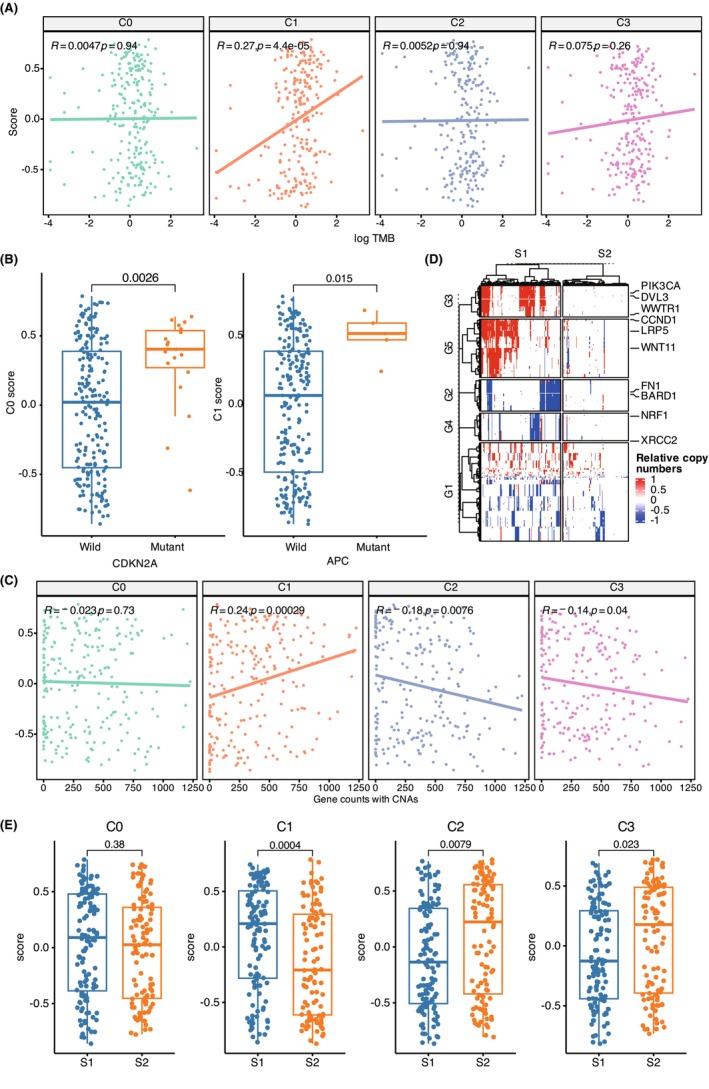
Association Between Tumour Cell Subpopulations and Genomic Alterations. (A) Examination of the correlation between point mutations and ssGSEA scores showed a positive correlation between C1 ssGSEA scores and tumour mutation burden (TMB). (B) Analysis of point mutations associated with C0 and C1 subpopulations revealed higher ssGSEA scores of C0 in samples with *CDKN2A* mutations and higher ssGSEA scores for C1 in samples with *APC* mutations, indicating a relationship between CDKN2A and APC dysregulation and the formation of C0 and C1 subpopulations. The *p*‐values were calculated by Student's *t*‐test. (C) Positive correlation between C1 ssGSEA scores and the number of genes with copy number alterations (CNAs), while C2 and C3 scores were negatively correlated with the number of genes with CNAs. C0 ssGSEA scores showed no significant correlation with CNAs. (D) Clustering of genes and tumour samples based on somatic copy number alteration (sCNA) profiles revealed five distinct gene sets (G1‐G5) representing chromosomal instability and stability. The *p*‐values were calculated by Student's *t*‐test. (E) The comparison of ssGSEA scores for tumor cell subpopulation markers between S1 and S2 subgroups.

In order to test whether the molecular features of tumour cell subpopulations are associated with genomic alterations, we separately examined the correlation between point mutations and copy number alterations (CNAs) with the ssGSEA scores of each tumour cell subpopulation. Firstly, we calculated the tumour mutation burden (TMB) for each sample in the TCGA cohort, and Pearson correlation analysis revealed a positive correlation between the ssGSEA scores of C1 and TMB, suggesting that the C1 subpopulation of tumour cells might accumulate more somatic mutations (Figure 4A). Next, we observed that samples with *CDKN2A* mutations had higher ssGSEA scores for C0, while samples with *APC* mutations had higher ssGSEA scores for C1, suggesting an association between dysregulation of *CDKN2A* and *APC* and their related pathways with the formation of C0 and C1 subpopulations. Furthermore, we calculated the number of genes with CNAs in each sample, and Pearson correlation analysis showed that the ssGSEA scores of C1 were positively correlated with the number of genes with CNAs, while the ssGSEA scores of C2 and C3 were negatively correlated with the number of genes with CNAs (*p* < 0.05). However, there was no significant correlation between the ssGSEA scores of C0 and the number of genes with CNAs. Additionally, we performed clustering of genes and tumour samples based on the somatic copy number alteration (sCNA) profile. This analysis revealed five distinct sCNA gene sets (G1‐G5), with representative mutated genes including *FN1* and *BARD1* deletions, *PIK3CA*, *DVL3*, *WWTR1* gains, *NRF1*, *XRCC2* deletions, and *CCND1*, *LRP5*, *WNT11* gains. Tumour samples were divided into two groups (S1 and S2) with significantly different chromosomal stability, represented by chromosomal instability and chromosomal stability, respectively. Similar to the Pearson correlation analysis, the ssGSEA scores of C1 were significantly higher in the chromosomal instability group (S1) compared to the chromosomal stability group (S2), while the ssGSEA scores of C2 and C3 were significantly lower in the strong chromosomal instability group (S1) compared to the chromosomal stability group (S2), and C0 showed no difference between the two groups (S1 and S2), suggesting that C1 exhibits higher chromosomal instability. These results indicated that the genomic variations, particularly point mutations and CNAs, are associated with the molecular characteristics of specific tumour cell subpopulations, suggesting a potential link between genomic alterations and the heterogeneity of tumour cells.

### 3.5 C0 subpopulation has a higher level of intercellular communications with fibroblasts and endothelial cells

**FIGURE 5:**
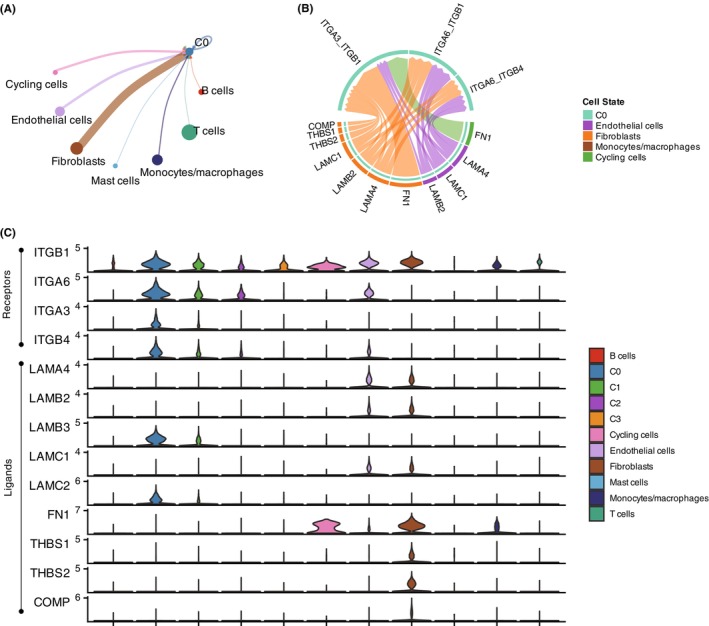
Intercellular Communication of Tumour Cell Subpopulations with Tumour Microenvironment (TME). (A) The signalling originating from cell types within TME to tumour cell subpopulations was predicted using CellChat. (B) Specific expression of receptors in the C0 subpopulation, including integrin‐a3b1, integrin‐a6b1 and integrin‐a6b4, while corresponding ligands like Laminins (LAMA4, LAMB2, LAMB3, LAMC1, LAMC2), Fibronectin (FN1), Thrombospondin (THBS1 and THBS2) and Cartilage Oligomeric Matrix Protein (COMP) were primarily expressed by fibroblasts and endothelial cells. (C) Expression patterns of receptors and ligands suggest enhanced intercellular communication between C0 and fibroblasts/endothelial cells within the tumour microenvironment.

In order to explore how different cell types in the tumour microenvironment interact with tumour cell subpopulations through intercellular communication, we used CellChat to establish a regulatory network between tumour cell subpopulations and various cell types in the tumour microenvironment. We found that different cell types in the tumour microenvironment exerted a stronger regulatory effect on the C0 subpopulation, with fibroblasts showing a higher probability of communication with C0 (Figure 5A). Notably, C0 exhibited specific expression of receptors, including integrin‐a3b1, integrin‐a6b1 and integrin‐a6b4, while their corresponding ligands included Laminins (LAMA4, LAMB2, LAMB3, LAMC1, LAMC2), Fibronectin (FN1), Thrombospondin (THBS1 and THBS2) and Cartilage Oligomeric Matrix Protein (COMP), which were primarily expressed by fibroblasts and endothelial cells (Figure 5B,C). These findings indicated that the C0 subpopulation has a higher level of intercellular communication with fibroblasts and endothelial cells within the tumour microenvironment.

### 3.6 The function of C0 subpopulation may be maintained through integrin/MAPK signalling pathway

**FIGURE 6:**
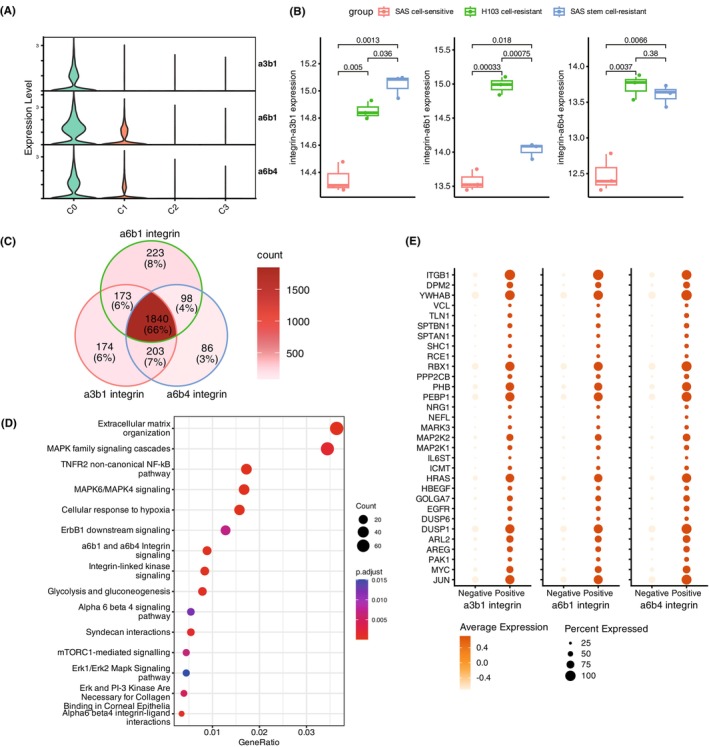
Role of Integrin Receptors in C0 Subpopulation Functionality. (A) Analysis of single‐cell transcriptome data revealed specific high expression of integrin receptors a3b1, a6b1 and a6b4 in the C0 subpopulation. (B) Gene expression profiles of three OSCC cell lines demonstrated higher expression of the three integrin receptors in drug‐resistant cell lines and drug‐resistant tumour stem cell lines compared to the sensitive cell line. Differential expression patterns were observed for a3b1 and a6b1 integrins between the two drug‐resistant cell lines. The *p*‐values were calculated by Student's *t*‐test. (C) Overlapping upregulated genes among tumour cells positive for the three integrin receptors, indicating a high degree of commonality. (D) Commonly upregulated genes in cells positive for the three integrin receptors were enriched in extracellular matrix organization, integrin‐related, and MAPK‐related pathways. (E) Upregulation of key regulatory genes in MAPK pathway among tumour cells positive for the three integrin receptors, suggesting integrin‐MAPK signalling pathway involvement in C0 subpopulation functionality.

Considering that the functional maintenance of the C0 cell subpopulation is highly correlated with the integrin receptors a3b1, a6b1 and a6b4, understanding the downstream regulatory pathways is crucial for elucidating the molecular mechanisms behind the functional maintenance of the C0 cell subpopulation. Through the analysis of tumour single cells, we found specific high expression of the three integrin receptors in the C0 cell subpopulation (Figure 6A). Furthermore, by comparing the gene expression profiles of three different OSCC (oral squamous cell carcinoma) cell lines, we observed that the three integrin receptors were all highly expressed in the drug‐resistant cell line and drug‐resistant tumour stem cell line compared to the sensitive cell line (Figure 6B). It was noteworthy that there were differences of a3b1 and a6b1 integrins between drug‐resistant cell lines and drug‐resistant tumour stem cell lines, whereas no significant difference existed for a6b4 integrin between the two drug‐resistant cell lines (Figure 6B), implying that a3b1 and a6b1 integrins potentially play distinct roles in the mechanisms of drug resistance, while a6b4 integrin may exert similar effects on both types of drug‐resistant cell lines.

Based on single‐cell transcriptome data, we compared the gene expression profiles of tumour cells that were positive and negative for the three integrin receptors, respectively. We found a high degree of overlap in the upregulated genes among tumour cells that were positive for the three integrin receptors (Figure 6C). The commonly upregulated genes in tumour cells positive for the three integrin receptors were primarily involved in extracellular matrix organization, integrin‐related pathways and MAPK‐related pathways (Figure 6D). Furthermore, key regulatory genes in the MAPK pathway, such as MAP2K1, MAP2K2 and SHC1, were upregulated in tumour cells positive for the three integrin receptors (Figure 6E). These results suggest that the functional maintenance of the C0 tumour cell subpopulation primarily relies on the integrin‐MAPK signalling pathway.

## 4 DISCUSSION

Oral squamous cell carcinoma (OSCC) is a prevalent malignancy characterized by its heterogeneity, aggressive behaviour and variable treatment outcomes. Recent research has significantly advanced our understanding of head and neck squamous cell carcinoma (HNSCC) by revealing the existence of distinct tumour cell subpopulations.^9^ Despite this progress, the clinical relevance of these subpopulations and the underlying mechanisms governing their behaviour in OSCC remain largely elusive. In this study, we identified eight major cell types, including B cells, cancer cells, cycling cells, endothelial cells, fibroblasts, mast cells, monocytes/macrophages and T cells. The identification of tumour cell subpopulations in OSCC using single‐cell transcriptomic data is a significant advancement in understanding tumour heterogeneity and microenvironment interactions.^22^ Consequently, we performed re‐clustering of the cancer cells and identified four different tumour cell subpopulations labelled as C0, C1, C2 and C3. These cell subpopulations exhibited unique gene expression patterns and molecular characteristics, suggesting the presence of intratumoral heterogeneity in OSCC. This finding aligns with previous research that has highlighted the importance of tumour cell subpopulations in cancer progression, treatment response and clinical outcomes.^23^, ^24^ Intratumoral heterogeneity is a well‐established phenomenon in various cancer types, and understanding the molecular features of different subpopulations is essential for devising targeted therapies and personalized treatment approaches.^25^

Characterizing the specific pathways and functions associated with each tumour cell subpopulation (e.g. collagen formation, cell cycle regulation and keratinization) provides valuable insights into the diverse roles that different subpopulations may play in tumour development and progression. This knowledge could potentially lead to the identification of biomarkers or therapeutic targets specific to each subpopulation, which could improve treatment outcomes in OSCC patients. Furthermore, the analysis of clinical characteristics and survival outcomes associated with tumour cell subpopulations underscores the clinical relevance of intratumoral heterogeneity. The observation that certain subpopulations (C0 and C1) are correlated with adverse prognosis and metastatic potential supports the idea that targeting specific subpopulations may be crucial for preventing disease progression and metastasis.^26^, ^27^ Additionally, the representative genes of C0 and C1 subpopulations were differentially expressed in drug‐resistant and sensitive cell lines, suggesting that the specific tumour cell subpopulations (C0 and C1) have different gene expression profiles and are associated with differential sensitivity to cisplatin treatment. These results not only suggest that OSCC patients with a higher proportion of the C1 subpopulation may benefit from chemotherapy, whereas those with a higher proportion of the C0 subpopulation might require an alternative therapy to prevent cisplatin‐based chemoresistance. They also emphasize the importance of taking into account the heterogeneity of cancer cell populations when studying drug responses and devising therapeutic strategies.^28^

It is well established that the accumulation of genetic variations can enhance tumour evolution and intratumoral heterogeneity.^29^, ^30^ The positive correlation between the ssGSEA scores of C1 and the tumour mutation burden (TMB) suggests that the C1 subpopulation of tumour cells might accumulate more somatic mutations. The association between C1 and TMB may have clinical implications, as high TMB has been associated with increased responsiveness to immunotherapies in certain cancer types.^31^ Furthermore, the specific genomic alterations, such as *CDKN2A* and *APC* mutations, associated with the formation of C0 and C1 subpopulations provide insight into the potential drivers of tumour cell heterogeneity. *CDKN2A* is a known tumour suppressor gene involved in cell cycle regulation,^32^ while APC is a critical component of the Wnt signalling pathway.^33^ The dysregulation of the two genes and their related pathways could contribute to the distinct molecular characteristics of C0 and C1 subpopulations. *CDKN2A* is a gene that regulates the cell cycle, and the loss of CDKN2A may lead to cell cycle progression. However, the relationship between *CDKN2A* loss and the epithelial‐mesenchymal transition (EMT) process in tumour cells has not been reported. *APC* is a crucial gene that regulates the WNT pathway, and the WNT pathway is closely associated with cell proliferation.^34^ Therefore, further experimental validation is needed to understand the relationship between mutations in these two genes and the two subpopulations of tumour cells. The correlation between the ssGSEA scores of C1 and the number of genes with copy number alterations (CNAs) suggests that the C1 subpopulation may exhibit higher chromosomal instability. Genomic instability, as indicated by CNAs, is a hallmark of cancer and is associated with tumour progression and drug resistance.^35^ The finding that C1 has a higher level of chromosomal instability aligns with previous research that has linked chromosomal instability to increased tumour aggressiveness and adverse clinical outcomes.^36^ These results highlight the association between the molecular characteristics of tumour cell subpopulations and genomic variations, particularly point mutations and CNAs, and provide valuable insights into the genetic drivers of tumour cell heterogeneity.

Furthermore, we also focus on exploring the intercellular communication between tumour cell subpopulations and various cell types in the tumour microenvironment in oral squamous cell carcinoma (OSCC). The finding that different cell types in the tumour microenvironment exert a stronger regulatory effect on the C0 subpopulation, with fibroblasts showing a higher probability of communication with C0, suggests that the C0 subpopulation may have a unique relationship with fibroblasts and endothelial cells. Intercellular communication between tumour cells and stromal cells in the tumour microenvironment plays a critical role in tumour progression, invasion and metastasis.^37^, ^38^, ^39^ The specific expression of receptors such as integrin‐a3b1, integrin‐a6b1 and integrin‐a6b4 on C0, along with their corresponding ligands primarily expressed by fibroblasts and endothelial cells, indicates potential crosstalk and signalling pathways involved in promoting the functional maintenance of the C0 subpopulation. Integrins, a family of cell adhesion receptors, play a significant role in cancer, including head and neck squamous cell carcinoma (HNSCC) and oral squamous cell carcinoma (OSCC). Integrins are involved in various stages of tumour progression, including cell proliferation, migration, invasion and metastasis.^40^ Moreover, integrins have been implicated in promoting drug resistance in cancer cells, leading to treatment failure,^41^ which facilitated the interactions between tumour cells and components of the tumour microenvironment, influencing tumour cell survival, invasion and immune evasion.

In addition, we investigated the potential mechanism behind the functional maintenance of the C0 tumour cell subpopulation in oral squamous cell carcinoma (OSCC) and found that the high expression of integrin receptors a3b1, a6b1 and a6b4 in the C0 subpopulation may be crucial for its functional maintenance. The upregulation of key regulatory genes in the MAPK pathway, such as MAP2K1, MAP2K2 and SHC1, in tumour cells positive for the three integrin receptors further supports the involvement of the integrin‐MAPK signalling pathway in the functional maintenance of the C0 subpopulation. The MAPK pathway is known to regulate various cellular processes, including cell proliferation, survival and migration,^42^, ^43^ which are critical for cancer cell growth and metastasis. The identification of the integrin‐MAPK signalling pathway as a potential mechanism behind the functional maintenance of the C0 tumour cell subpopulation provides valuable insights into the molecular basis of tumour heterogeneity and drug resistance. Targeting the integrin‐MAPK pathway may present new therapeutic opportunities for OSCC treatment, especially for drug‐resistant tumour subpopulations. Unfortunately, the targeted drugs for integrin receptors a3b1, a6b1 and a6b4 have not undergone clinical trials or been incorporated into clinical practice. In contrast, MEK1/2 inhibitors such as Binimetinib and Cobimetinib have received approval for the treatment of melanoma,^44^ presenting a potential alternative therapy for OSCC.

However, the present study does have some limitations. Firstly, molecular characteristics of the cancer cell subpopulations need to be confirmed through molecular experiments. Secondly, the association between the cancer cell subpopulations with chemotherapeutic response should be validated in a larger OSCC cohort. In summary, the present study provides a comprehensive analysis of OSCC subpopulations by analysing their clinical relevance and functional characteristics, which is valuable for precision medicine in OSCC.

## AUTHOR CONTRIBUTIONS

**Jia Li:** Data curation (equal); methodology (equal); software (equal); writing – original draft (equal). **Shengjiao Li:** Methodology (equal); resources (equal); software (equal). **Mingyang Shu:** Formal analysis (equal); investigation (equal); resources (equal). **Weiwei Hu:** Conceptualization (equal); funding acquisition (equal); software (equal); visualization (equal); writing – original draft (equal); writing – review and editing (equal).

## FUNDING INFORMATION

This work was supported by the Shanghai Municipal Natural Science Foundation (No. 22ZR1467200).

## CONFLICT OF INTEREST STATEMENT

The authors have declared that there are no competing interests.

## Supporting information


Table S1.

Table S2.

Table S3.

Data S1.

## Data Availability

The gene expression and clinical data used to support this study are available at the UCSC Xena (TCGA, http://xena.ucsc.edu/), ArrayExpress (http://www.ebi.ac.uk/biostudies/arrayexpress) databases. The related studies (and datasets) have been cited within the text as references.
