# Supplementary material for: Unravelling the heterogeneity of oral squamous cell carcinoma by integrative analysis of single‐cell and bulk transcriptome data
Source: J Cell Mol Med. 2024 Jan 26;28(3):e18108. doi: 10.1111/jcmm.18108 (PMC10844683; doi:10.1111/jcmm.18108)
Supplement: Supplementary file 4 — Data S1. [file JCMM-28-e18108-s001.docx]

Thanks for your e-mail. The captions for Tables S1-S3 are listed as follows:

Table S1. The differentially expressed genes of cell types in OSCC by scRNA-seq

Table S2. The differentially expressed genes of the cancer cell subpopulations in OSCC by scRNA-seq

Table S3. The pathways enriched by the differentially expressed genes of the cancer cell subpopulations.
